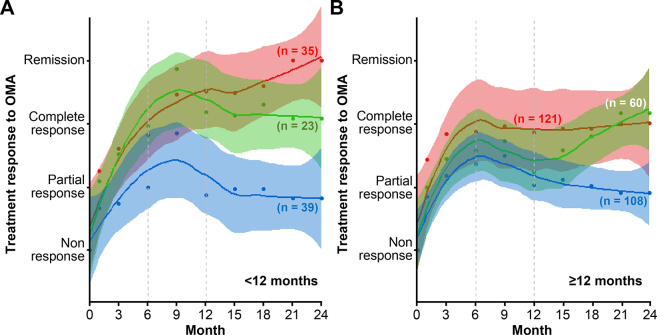# Predicting responses to omalizumab in antihistamine-refractory chronic urticaria: A real-world longitudinal study

**DOI:** 10.1016/j.jacig.2024.100245

**Published:** 2024-03-19

**Authors:** Hyun-Young Lee, Hyun-Seob Jeon, Jae-Hyuk Jang, Youngsoo Lee, Yoo Seob Shin, Dong-Ho Nahm, Hae-Sim Park, Young-Min Ye

**Affiliations:** a Clinical Trial Center, Ajou University Medical Center, Suwon, Korea; b Department of Allergy and Clinical Immunology, Ajou University School of Medicine, Suwon, Korea

**Keywords:** Chronic urticaria, omalizumab, treatment response, predictor, total IgE

## Abstract

**Background:**

Treating chronic urticaria (CU) that is unresponsive to H1-antihistamines (H1AHs) is challenging, and the real-world effectiveness of omalizumab remains unclear.

**Objective:**

Our aim was to evaluate the real-world effectiveness of omalizumab, optimal response assessment timing, and predictive factors.

**Methods:**

Initially, 5535 patients with CU who were receiving at least 20 mg of loratadine daily for at least 6 months (January 2007-August 2021) were screened. Ultimately, 386 patients who had been receiving omalizumab add-on treatment for >6 months were followed-up for more than 2 years. Predictors of treatment response to omalizumab add-on therapy for patients with antihistamine-refractory CU were identified by using a generalized linear model.

**Results:**

In our retrospective cohort, omalizumab treatment showed cumulative response rates of 55.2% at 3 months, 71.0% at 6 months, and 81.4% at 9 months for patients with H1AH-refractory CU. Analysis of longitudinal responses to omalizumab treatment revealed 3 distinct clusters: favorable (cluster 1 [n = 158]), intermediate (cluster 2 [n =1 43]), and poor responses (cluster 3 [n = 85]). Subjects were categorized on the basis of whether they had achieved a complete response within 3 months; 213 early responders, 117 late responders, and 56 nonresponders were identified. The initial dose of omalizumab differed significantly among the 3 clusters. Low total IgE level (<40 kU/L) predicted nonresponse (odds ratio [OR] = 3.10 [*P* = .018]). Early responders were associated with a higher initial omalizumab dose (≥300 mg) (OR = 2.07 [*P* = .016]), higher basophil counts (OR = 2.0 [*P* = .014]), total IgE levels exceeding 798 kU/L (OR = 0.37 [*P* = .047]), and lower platelet-to-lymphocyte ratio (OR = 0.50 [*P* = .050]).

**Conclusion:**

Real-world data reveal 3 distinct clusters for response to omalizumab treatment; confirm low serum total IgE level (<40 kU/L) as a predictor of nonresponse; and identify potential biomarkers, including IgE level, basophil count, and PLR, for early responders.

Chronic urticaria (CU) is a prevalent skin disorder characterized by the recurrence of wheals and/or angioedema persisting for more than 6 weeks; it is managed primarily by using H1-antihistamines (H1AHs).^1^ Nonetheless, approximately 50% of patients with CU continue to exhibit symptoms even after a 4-fold increase in H1AH dose.^2^^,^^3^ Some patients experience a poor quality of life, especially those experiencing heightened disease activity and uncontrolled CU despite conventional treatment.^4^ For patients with antihistamine-refractory chronic spontaneous urticaria (CSU) whose symptoms remain uncontrolled despite receiving high-dose H1AH treatment, contemplating the addition of immunomodulating agents such as omalizumab, cyclosporin, dapsone, hydroxychloroquine, and methotrexate may be pertinent. Mast cells play a pivotal role in the pathogenesis of CSU, primarily through persistent activation of the IgE receptor pathway. Additionally, recent research has spotlighted additional receptors and signaling pathways contributing to this process.^5^

Omalizumab, a recombinant humanized IgG1 mAb that targets IgE, is recognized for its ability to decrease serum-free IgE levels and suppress FcεRIα expression in mast cells and other inflammatory cells.^6^ Both clinical trials and real-world studies have affirmed the efficacy of omalizumab in treating CU, rendering it the sole licensed treatment.^7^^,^^8^ Recent international guidelines underscore omalizumab as the principal option for patients with H1AH-refractory CSU.^1^ However, this drug does not elicit uniform responses across all patients. Approximately 60% of patients observe positive outcomes within 6 months, whereas around 15% exhibit no response, even with doses up to 600 mg.^9^ Consequently, questions regarding the optimal timing and methodology for assessing omalizumab treatment response in patients with CSU are frequently posited by both physicians and patients. Moreover, considering the cost of omalizumab treatment, addressing how to identify the subgroup of patients with CSU who are unlikely to respond to omalizumab add-on therapy before its initiation becomes paramount. In a previous study of patients with CSU, the percentage of complete responders increased steadily as dosing persisted over a 24-week active treatment period.^10^ Although previous studies have noted associations between the omalizumab response and factors such as basophil FcεRI expression, baseline IgE levels, and the presence of autoantibodies,^11^, ^12^, ^13^ whether these factors can robustly predict the omalizumab response in patients with CSU remains to be distinctly determined .

Using a longitudinal cohort of patients with H1AH-refractory CSU who underwent omalizumab treatment for more than 6 months, our study aimed to categorize the patients on the basis of their responses to omalizumab, assess the validity of the resulting clusters by analyzing changes in medication scores and the time to achieve a complete response, and identify predictors of nonresponders and late responders within these clusters.

## Methods

### Study design

We conducted a longitudinal retrospective cohort study analyzing patients with CU at the allergy and clinical immunology department of a tertiary university hospital to which all of the patients were referred by primary health care providers in Korea between August 2007 and December 2021. Data were extracted from the electronic medical records of individuals diagnosed with CU, using the L50 code from the 10th revision of the *International Classification of Diseases*. This included details on prescribed CU medications, diagnostic information, laboratory test results, and visit history. This study received approval from the ethical review board of Ajou University Hospital (approval no. AJIRB-MED-SMP-19-332).

### Study population

**Fig 1:**
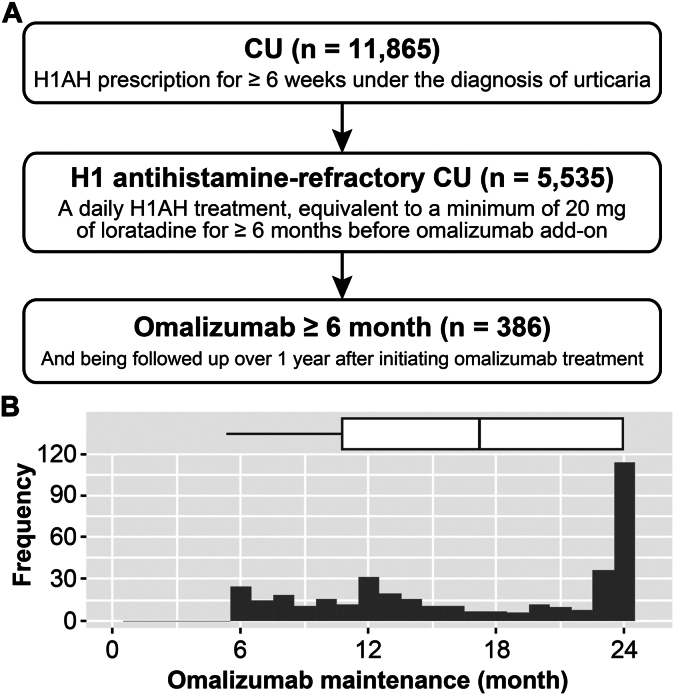
Consolidated Standards of Reporting Trials flow of the study subjects (**A**) and distribution of omalizumab treatment duration (**B**). Box plot illustrates the mean and interquartile range of omalizumab treatment duration.

From an initial cohort of 11,865 individuals prescribed H1AH for more than 6 weeks as part of their primary urticaria diagnosis, we selected 5,533 patients with H1AH-refractory CU (Fig 1). These patients had been receiving daily H1AH treatment, equivalent to at least 20 mg of loratadine, for a minimum of 6 months before starting omalizumab add-on therapy. Of the total 5533 patients with H1AH-refractory CU, 904 had received omalizumab at least once. Among those 904 patients, our analysis focused on 386 subjects who had received omalizumab for a minimum of 6 months and consistently attended outpatient visits for at least 12 months after initiating omalizumab add-on therapy. The index date was defined as the date of the first omalizumab prescription. This retrospective cohort study included prescription records spanning from 6 months before the index date to 2 years after the index date.

### Classification of treatment responses to omalizumab add-on therapy

**Fig 2:**
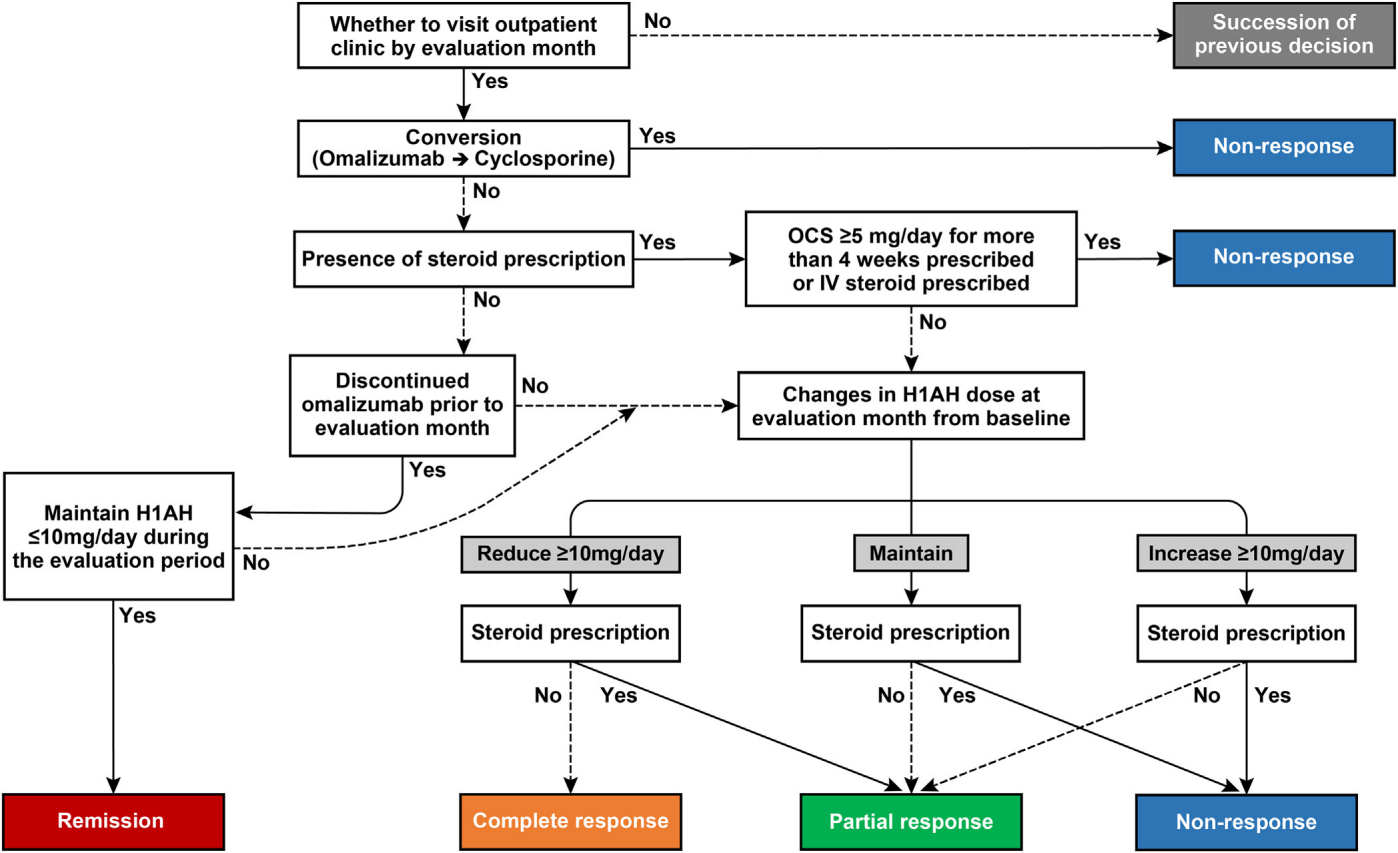
Decision flow for classifying treatment response to omalizumab. OCS as an equivalent dose of prednisolone; H1AH as an equivalent dose of loratadine. *IV,* Intravenous.

After the patients had started receiving omalizumab add-on therapy, their responses were categorized into 4 groups: remission, complete response, partial response, and nonresponse, based on medication changes (Fig 2). Nonresponse was defined as meeting any of the following criteria: transitioning from omalizumab to cyclosporine or methotrexate, receiving oral corticosteroids (OCSs) at a daily dose of 5 mg or higher for more than 4 weeks, or receiving any prescription for intravenous steroids. Remission was identified when patients no longer needed H1AH maintenance or when their H1AH prescription was reduced to less than 10 mg of loratadine equivalent after discontinuation of omalizumab. A complete response was defined as reduction of the H1AH dose by at least 10 mg of loratadine equivalent without an OCS prescription while omalizumab therapy was maintained. Partial responses were defined as those cases in which the H1AH dose was either kept stable or increased without an OCS prescription following initiation of omalizumab add-on therapy or an OCS prescription was introduced despite a reduction in H1AH dose. The assessment of treatment responses to omalizumab add-on therapy was conducted at 1, 3, 6, 9, 12, 15, 18, 21, and 24 months after the index date for each subject. To investigate whether these clusters were influenced by the time taken to achieve a complete response, we further categorized responders as either early or late responders. Early responders were defined as individuals who achieved remission or a complete response within 3 months following the initiation of omalizumab treatment.

### Medication score assessment

To validate the trajectories, medication scores were used for each prescription, excluding omalizumab, as described in a previous report.^14^ The daily dose of H1AH was converted to an equivalent loratadine dose of 10 mg. A score of 1 was attributed to H1AH doses of 10 mg per day. OCS daily doses were converted to an equivalent dose of prednisolone, with scores of 5, 10, and 15 assigned to doses of less than 11 mg per day, 11 to 25 mg per day, and more than 25 mg per day, respectively. Furthermore, the use of cyclosporine was assigned a score of 8 points, leukotriene receptor antagonists received 2 points, and histamine-2 receptor antagonist use was assigned 2 points.^14^^,^^15^ Individuals devoid of medication records for CU over a 1-year period, despite hospital visits for other conditions, were assigned a medication score of 0 points during the follow-up period.

### Laboratory markers

Serum levels of total IgE and IgE specific to house dust mites were measured using the ImmunoCAP system (Thermo Fisher Scientific/Phadia, Uppsala, Sweden). A complete blood count and differential leukocyte count at the diagnosis of CU for each patient were collected. Basophil, eosinophil, lymphocyte, monocyte, and neutrophil counts were calculated by multiplying the white blood cell count by each differential ratio. Basopenia and eosinopenia were defined as less than 10/μL and less than 50/μL, respectively.^16^ The platelet-to-lymphocyte ratio (PLR) was determined by dividing the absolute platelet counts by the absolute lymphocyte count within the peripheral blood. A systemic inflammation response index (SIRI) was calculated by using the formula: (neutrophil count × monocyte count)/lymphocyte count.^17^

### Statistical analysis

To compare clinical characteristics among the 3 clusters and among early responders, late responders, and nonresponders, ANOVA was used for continuous parameters, and the Pearson chi-square test was used for categoric parameters. The K-means method, using the shape-respecting distance approach, was applied to cluster responses by using the R package KmlShape.^18^ To analyze the time to achieve the first complete response following omalizumab add-on treatment, a Kaplan-Meier plot with a log-rank test was used. Moreover, agreement between the 3 treatment response clusters and the 3 groups categorized based on time to a complete response was evaluated using the Fleiss κ. To visualize changes in treatment response over the 24-month period, an alluvial plot was used.

To identify predictors of the treatment response to omalizumab add-on therapy in patients with H1AH-refractory CU, a generalized linear model (GLM) with a logit link function was applied. The forest plot illustrated the odds ratios (ORs) along with 95% CIs obtained from the multivariate analyses of the GLM. A vertical line indicating no effect (OR = 1) was also included in the plot. Significance was determined at *P* values less than .05. All statistical analyses were conducted by using R, version 4.1.0 software (R Development Core Team [http://www.r-project.org]).

## Results

### Clinical characteristics of the study subjects

Among the 386 patients with H1AH-refractory CU meeting the criteria of receiving omalizumab treatment for at least 6 months and being followed up for more than 1 year, 152 (39.4%) were female. The mean age of the patients was 41.3 years (Table I). We found that 18, 5, 17, 3, and 3 patients had allergic rhinitis, asthma, atopic dermatitis, food allergy, and drug allergy as their secondary disease code, respectively. No significant difference in the prevalence of these comorbidities among the 3 clusters and responder groups (Tables I and II). Omalizumab treatment was continued for an average of 16.9 months, with 37% of patients initiating treatment with a dose of 300 mg. Among the initial 243 patients who did not receive omalizumab in a dose of at least 300 mg at the beginning of treatment, 83 were subsequently found to have their doses increased to 300 mg or more at least once after the second month of omalizumab add-on therapy (Table I). Overall, 289 patients (74.9%) received omalizumab treatment for a period exceeding 12 months.

**Table I:** Clinical characteristics of the members of the 3 clusters

Characteristic	Total (n = 386)	Cluster 1 (n = 158)	Cluster 2 (n = 143)	Cluster3 (n = 85)	*P* value
Female sex, no. (%)	152 (39.4%)	69 (43.7%)	49 (34.3%)	34 (40.0%)	.247
Age at diagnosis (y), mean ± SD	41.3 ± 13.1	42.0 ± 13.3	41.6 ± 12.9	39.6 ± 13.3	.199
Age at index date (y), mean ± SD	42.1 ± 13.0	42.6 ± 13.0	42.6 ± 13.0	40.4 ± 12.8	.272
Secondary disease code, no. (%)					
J30: Allergic rhinitis	18 (4.7%)	7 (4.4%)	7 (4.9%)	4 (4.7%)	1.000
J45: Asthma	5 (1.3%)	2 (1.3%)	1 (0.7%)	2 (2.4%)	.547
L20: Atopic dermatitis	17 (4.4%)	9 (5.7%)	5 (3.5%)	3 (3.5%)	.660
T78.1: Food allergy	3 (0.8%)	3 (1.9%)	0	0	.241
T88.7: Drug allergy	3 (0.8%)	1 (0.6%)	0	2 (2.4%)	.174
Starting dose of OMA ≥ 300 mg, no. (%)	143 (37.0%)	71 (44.9%)	44 (30.8%)	28 (32.9%)	.027
Receiving OMA in a dose of ≥300 mg at least once after the second month, no. (%)	83 (34.2%)	23 (26.4%)	35 (35.4%)	25 (43.9%)	.094
Duration of OMA maintenance (mo), mean ± SD	16.9 ± 6.6	16.5 ± 6.7	18.7 ± 5.7	14.8 ± 6.9	.266
Intervals of OMA in 6-mo period (no.), mean ± SD	1.7 ± 0.7	1.7 ± 0.7	1.8 ± 0.7	1.6 ± 0.6	.263
Duration of urticaria treatment (mo), mean ± SD	23.0 ± 2.5	22.5 ± 3.0	23.3 ± 2.0	23.4 ± 1.9	.003
BMI (kg/m^2^), mean ± SD	23.9 ± 3.5	23.8 ± 3.3	24.0 ± 3.8	23.8 ± 3.3	.909
Combined angioedema, no. (%)	101 (26.2%)	45 (28.5%)	39 (27.3%)	17 (20.0%)	.333
HDM sIgE level < 0.35 kU/L, n of n (%)	151/325 (46.5%)	65/135 (48.1%)	60/124 (48.4%)	26/66 (39.4%)	.435
Basopenia, n of n (%)	14/346 (4.0%)	5/143 (3.5%)	5/126 (4.0%)	4/77 (5.2%)	.829
Eosinopenia, n of n (%)	69/346 (19.9%)	31/143 (21.7%)	21/126 (16.7%)	17/77 (22.1%)	.513
Total IgE level (kU/L), mean ± SD	314.6 ± 423.2	306.1 ± 401.2	258.4 ± 312.1	430.4 ± 588.0	.099
Total IgE level < 40 kU/L, n of n (%)	36/366 (9.8%)	14/153 (9.2%)	12/136 (8.8%)	10/77 (13.0%)	.577
SIRI, mean ± SD	1.4 ± 2.2	1.4 ± 1.4	1.2 ± 1.0	1.7 ± 4.0	.447
PLR, mean ± SD	10.4 ± 7.6	10.5 ± 6.2	10.1 ± 7.6	10.7 ± 9.8	.960
Basophil count (/μL), mean ± SD	36.0 ± 20.2	35.0 ± 18.4	36.3 ± 22.5	37.6 ± 19.3	.350
Eosinophil count (/μL), mean ± SD	158.4 ± 174.3	157.7 ± 182.7	159.8 ± 186.4	157.4 ± 135.3	.991
Lymphocyte count (10³/μL), mean ± SD	2.2 ± 0.8	2.2 ± 0.8	2.2 ± 0.7	2.1 ± 0.8	.673
Monocyte count (10³/μL), mean ± SD	0.5 ± 0.2	0.5 ± 0.2	0.5 ± 0.2	0.6 ± 0.3	.731
Neutrophil count (10³/μL), mean ± SD	4.8 ± 2.7	4.8 ± 2.6	4.7 ± 2.4	4.7 ± 3.2	.682
Platelet count (10³/μL), mean ± SD	21.1 ± 10.9	21.4 ± 10.6	20.6 ± 9.8	21.1 ± 13.1	.758
WBC count (10³/μL). mean ± SD	7.7 ± 2.9	7.8 ± 2.9	7.7 ± 2.6	7.6 ± 3.5	.601
C3 (mg/dL), mean ± SD	114.1 ± 19.6	115.7 ± 20.1	113.5 ± 20.3	112.0 ± 16.9	.190
C4 (mg/dL), mean ± SD	26.6 ± 8.4	27.3 ± 8.5	26.9 ± 8.3	24.8 ± 8.2	.077

*BMI*, Body mass index; *C3*, complement 3; *C4*, complement 4; *HDM*, house dust mite; *OMA*, omalizumab; *sIgE*, specific IgE; *WBC*, white blood count.

**Table II:** Comparison of clinical parameters according to the 3 responder groups

Parameter	Early responders (n = 213)	Late responders (n = 117)	Nonresponders (n = 56)	*P* value
Female sex, no. (%)	83 (39.0%)	46 (39.3%)	23 (41.1%)	.960
Age at diagnosis (y), mean ± SD	41.1 ± 13.3	42.9 ± 12.8	38.6 ± 13.1	.131
Age at index date (y), mean ± SD	41.8 ± 13.0	43.9 ± 12.9	39.5 ± 12.6	.101
Secondary disease code, no. (%)				
J30: Allergic rhinitis	13 (6.1%)	3 (2.6%)	2 (3.6%)	.365
J45: Asthma	4 (1.9%)	0	1 (1.8%)	.348
L20: Atopic dermatitis	11 (5.2%)	3 (2.6%)	3 (5.4%)	.512
T78.1: Food allergy	3 (1.4%)	0	0	.722
T88.7: Drug allergy	1 (0.5%)	1 (0.5%)	1 (1.8%)	.403
Starting dose OMA ≥ 300 mg, no. (%)	88 (41.3%)	32 (27.4%)	23 (41.1%)	.034
Receiving OMA in a dose of ≥300 mg at least once after the second month, no. (%)	30 (24.0%)	39 (45.9%)	14 (42.4%)	.003
Duration of OMA maintenance (mo), mean ± SD	17.9 ± 6.3	15.0 ± 6.9	17.4 ± 6.1	<.001
Intervals of OMA in 6-mo period, mean ± SD	1.8 ± 0.8	1.6 ± 0.5	1.6 ± 0.7	.015
Duration of urticaria treatment (mo), mean ± SD	22.6 ± 2.8	23.5 ± 1.9	23.4 ± 2.1	.006
BMI (kg/m^2^), mean ± SD	23.8 ± 3.3	24.1 ± 4.0	23.8 ± 3.0	.806
Combined angioedema, no. (%)	54 (25.4%)	37 (31.6%)	10 (17.9%)	.144
HDM sIgE level < 0.35 kU/L, n of n (%)	89/190 (46.8%)	39/91 (42.9%)	23/44 (52.3%)	.582
Basopenia, n of n (%)	3/191 (1.6%)	9/104 (8.7%)	2/51 (3.9%)	.013
Eosinopenia, n of n (%)	33/191 (17.3%)	25/104 (24.0%)	11/51 (21.6%)	.363
Total IgE level (kU/L), mean ± SD	274.6 ± 371.5	352.6 ± 409.4	395.2 ± 599.9	.100
Total IgE level < 40 kU/L, n of n (%)	17/207 (8.2%)	9/107 (8.4%)	10/52 (19.2%)	.049
SIRI, mean ± SD	1.2 ± 1.1	1.3 ± 1.4	2.0 ± 4.9	.095
PLR, mean ± SD	10.1 ± 6.8	10.4 ± 6.4	11.4 ± 11.7	.579
Basophil count (/μL), mean ± SD	35.1 ± 17.5	36.6 ± 24.8	38.3 ± 19.2	.564
Eosinophil count (/μL), mean ± SD	147.2 ± 132.4	170.8 ± 239.3	175.1 ± 154.5	.410
Lymphocyte count (10³/μL), mean ± SD	2.2 ± 0.8	2.1 ± 0.7	2.1 ± 0.8	.411
Monocyte count (10³/μL), mean ± SD	0.5 ± 0.2	0.5 ± 0.2	0.6 ± 0.3	.214
Neutrophil count (10³/μL), mean ± SD	4.7 ± 2.3	4.9 ± 2.8	4.9 ± 3.6	.758
Platelet count (10³/μL), mean ± SD	20.9 ± 10.0	20.8 ± 10.2	22.2 ± 14.7	.717
WBC count (10³/μL), mean ± SD	7.7 ± 2.6	7.8 ± 3.0	7.9 ± 3.9	.923
C3 level (mg/dL), mean ± SD	115.1 ± 20.1	113.2 ± 19.6	111.7 ± 17.1	.521
C4 level (mg/dL), mean ± SD	27.7 ± 8.3	25.2 ± 8.5	24.9 ± 8.2	.026

*BMI*, Body mass index; *C3*, complement 3; *C4*, complement 4; *HDM*, house dust mite; *OMA*, omalizumab; *sIgE*, specific IgE; *WBC*, white blood count.

### Clusters of longitudinal treatment response trajectories

**Fig 3:**
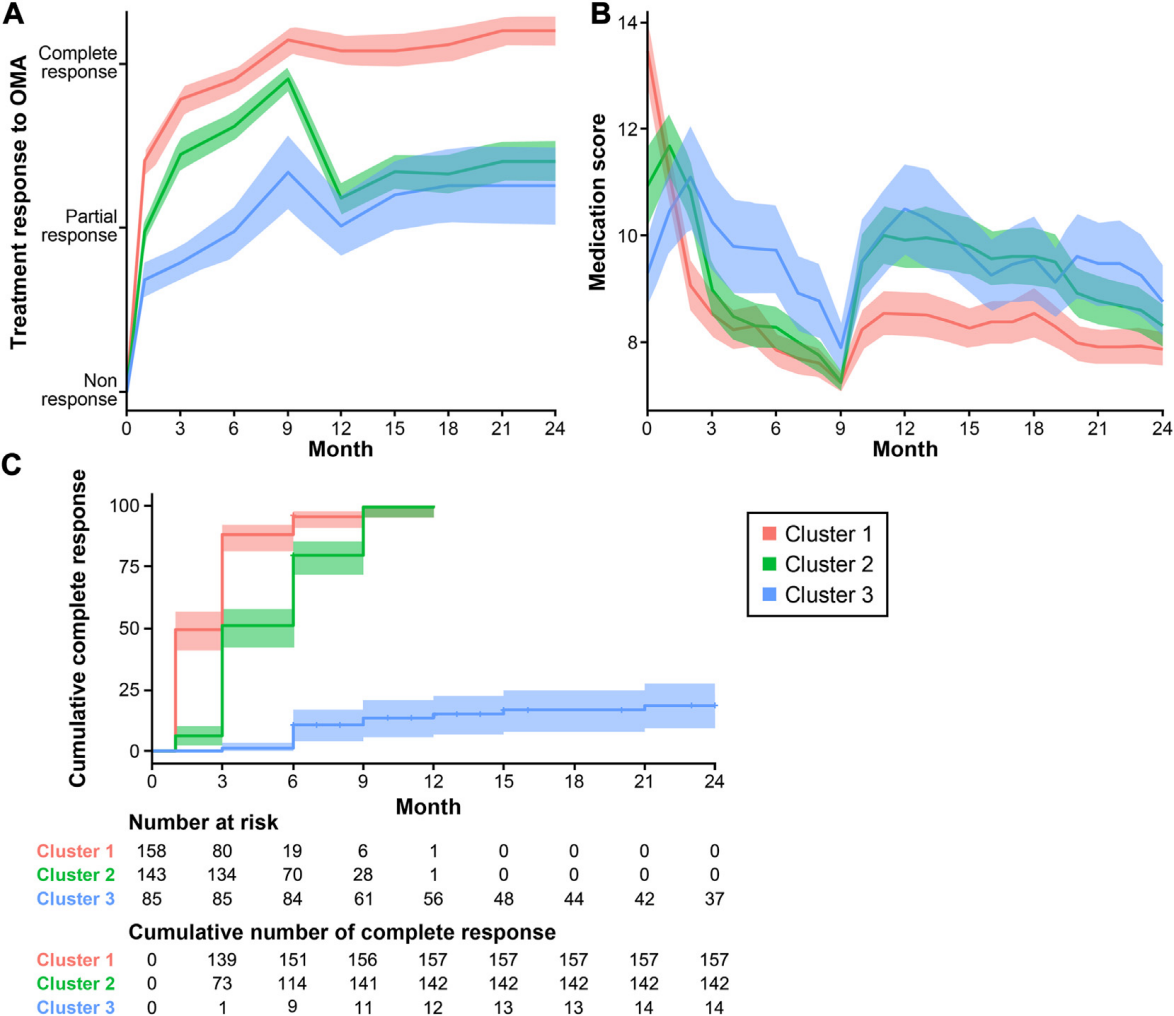
Generalized additive models with integrated smoothness estimation of longitudinal trajectory clusters of treatment response to omalizumab add-on therapy (**A**) and changes in medication scores during omalizumab treatment (**B**). **C,** Kaplan-Meier plot with a log-rank test depicts cumulative complete response to omalizumab add-on treatment among the 3 clusters. *OMA*, Omalizumab.

Three distinct clusters of longitudinal treatment response trajectories during omalizumab add-on therapy were identified (Fig 3): favorable responders (cluster 1 [n = 158]) demonstrated a consistently improved response following omalizumab treatment; intermediate responders (cluster 2 [n = 143]) exhibited a fluctuating response; and poor responders (cluster 3 [n = 85]) did not achieve a complete response.

Changes in medication scores among the 3 clusters over the course of omalizumab add-on treatment mirrored the variation in treatment responses over time (Fig 3). Among the patients in cluster 2, 9 months of treatment emerged as a significant time point, revealing distinct changes in medication scores. Within the initial 9 months of add-on therapy, medication scores decreased to levels comparable to those of cluster 1. Beyond this period, however, the pattern of change in medication scores resembled that seen in cluster 3 (poor response). In a comparison of the discontinuation rates within the first 9 months among the clusters, cluster 2 exhibited the lowest rate (5.6% [*P* < .001]). Moreover, the mean intervals between omalizumab injections did not differ significantly among the clusters. These findings imply that early discontinuation of treatment and extended intervals between omalizumab injections did not cause the varied treatment responses observed.

No significant differences in age, sex, duration of omalizumab treatment, or concomitant angioedema were found among the clusters. However, cluster 1 had higher medication doses in the 6 months before omalizumab treatment and also had more frequent initiation of omalizumab at a dose of at least 300 mg. However, the patients in cluster 1 received omalizumab at a dose of 300 mg or more at least once after the second month of omalizumab add-on therapy less frequently than did the patients in clusters 2 and 3, although this difference was not statistically significant. The time to achieve a complete response or urticaria remission differed significantly among the clusters (*P* < .001). At 3 months, complete response and/or remission rates were 80.0% for cluster 1, 51.0% for cluster 2, and 1.2% for cluster 3. Cluster 2 showed gradual improvement, reaching 98.6% by the ninth month. Early discontinuation and injection intervals did not influence response.

### Comparison of clinical characteristics among the 3 responder groups: early responders, late responders, and nonresponders

According to survival analyses, for patients with H1AH-refractory CU, the 3 month-period after first initiating omalizumab add-on therapy was identified as the optimal time frame for distinguishing early responders from late responders or nonresponders. We identified early responders (n = 213), late responders (n = 117), and nonresponders (n = 56) among patients with H1AH-refractory CU on the basis of achievement of a complete response within 3 months of initiating omalizumab add-on therapy. There were no significant differences in sex, age, or accompanied angioedema among these groups.

However, late responders had a shorter mean treatment duration (15.0 ± 6.9 months [*P* < .001]) than did early (17.9 ± 6.3 months) and nonresponders (17.4 ± 6.1 months). Early responders also had longer intervals between omalizumab injections. A higher proportion of patients in the late responder and nonresponder groups had received omalizumab in a dose of 300 mg or more at least once after the second month of omalizumab add-on therapy. This suggests that even with higher doses of omalizumab, some patients exhibited a poor response to omalizumab. Total IgE levels showed no significant differences, but the nonresponder group included more patients with low total IgE levels (ie, <40 kU/L [19.2%]) (*P* = .049). Basopenia frequency and complement 4 levels varied among the groups.

**Fig 4:**
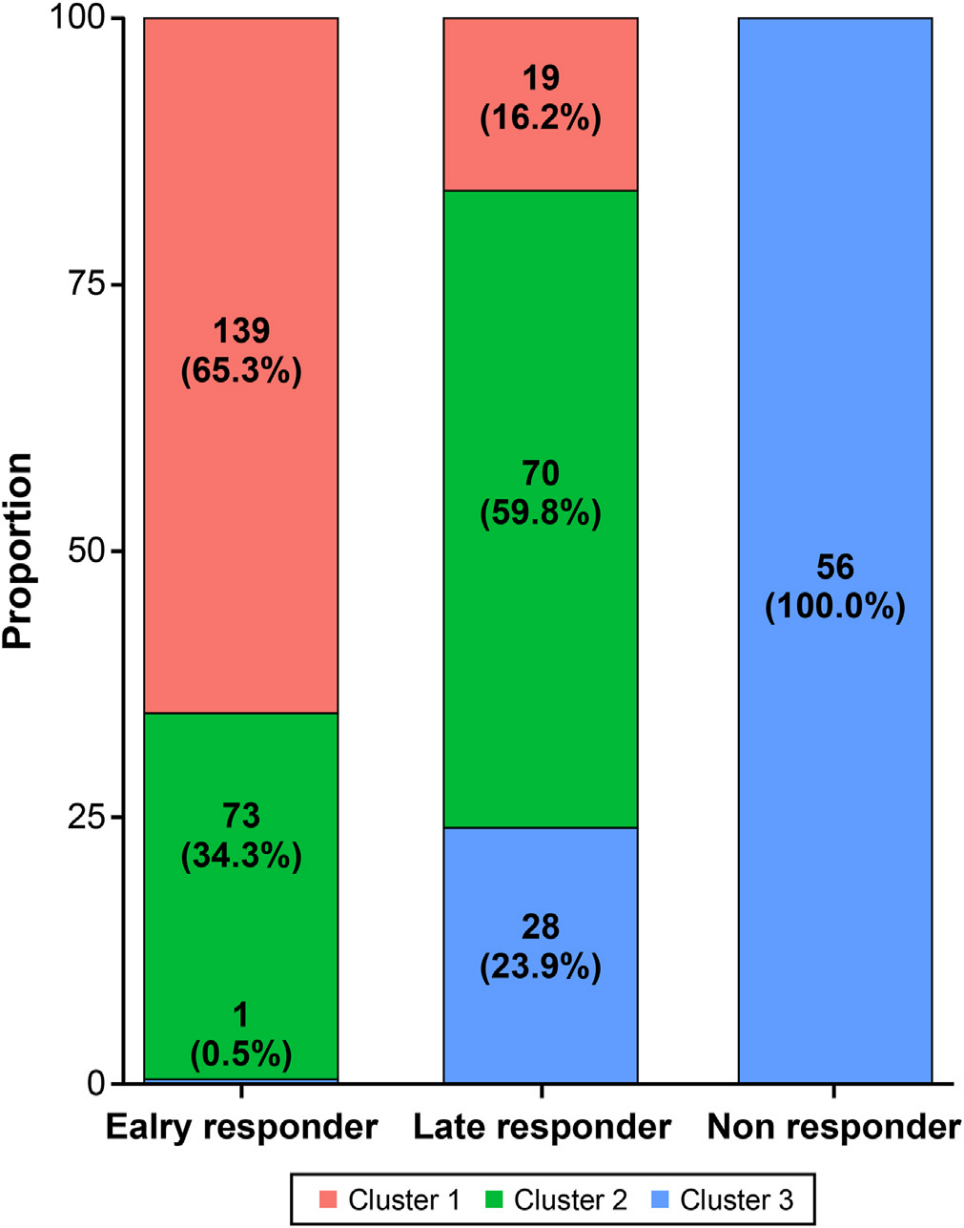
Proportions of the 3 clusters within early responders, late responders, and nonresponders.

Alluvial plots were used to display the proportions of the 3 clusters within the early responder, late responder, and nonresponder groups (Fig 4). The agreement between the 3 trajectory clusters and the response timing classifications was significant (Fleiss κ = 0.239 [*P* < .001]). Most of the early responders (55.2%) were in cluster 1, which also included fewer late responders (23.9%). All of the nonresponders were in cluster 3.

We also subdivided the trajectories on the basis of duration of omalizumab maintenance therapy (<1 year vs >1 year). Cluster 2 curves plateaued from 12 to 24 months for patients who discontinued omalizumab therapy within 12 months, but they gradually increased for those continuing omalizumab therapy beyond 12 months (see Fig E1 in the Online Repository at www.jaci-global.org).

### Predictors of the treatment response to omalizumab add-on therapy

**Fig 5:**
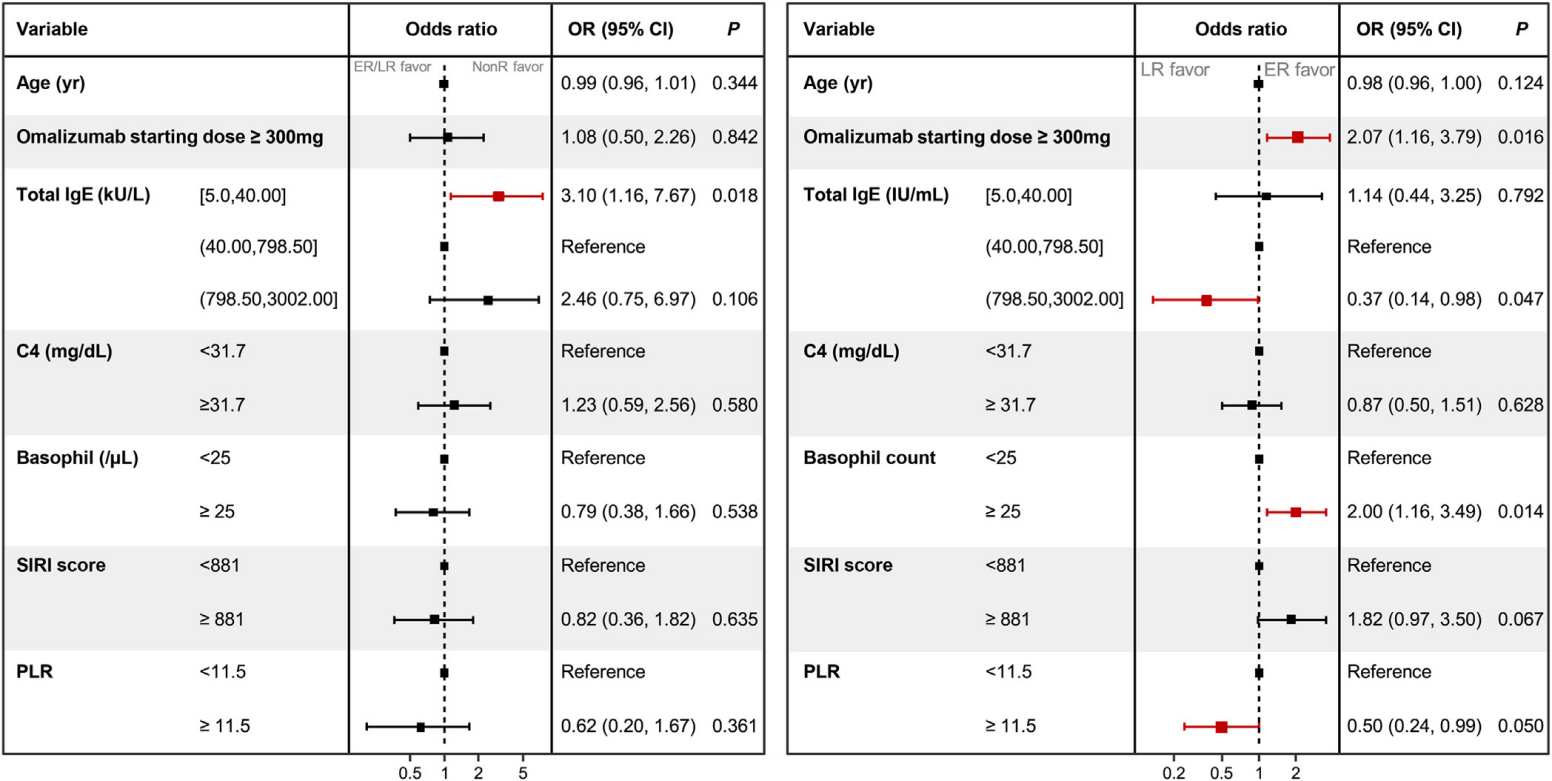
GLMs with logit link function to identify predictors of treatment response to omalizumab add-on therapy for patients with H1AH-refractory CU. *C4*, Complement 4; *ER*, Early responder; *LR*, late responder; *NR*, nonresponder.

To identify predictors of the treatment response to omalizumab add-on therapy for patients with H1AH-refractory CU, we conducted a multivariate analysis using a model that included age, a starting dose of omalizumab of at least 300 mg, total IgE level, complement 4 levels, basophil counts, SIRI scores, and PLR. Total IgE levels were categorized into the upper and lower 10th percentiles; the cutoff values for other laboratory markers were set at the medians of our study population. A GLM with a logit link function revealed significant associations between being a nonresponder and having a low total IgE level (<40 kU/L [OR = 3.10; 95% CI = 1.16-7.67 (*P* = .018)] [Fig 5]). Among those individuals who responded positively to omalizumab add-on therapy, significant predictors for being an early responder included initiating omalizumab therapy at a dose of 300 mg or higher (OR = 2.07; 95% CI = 1.16-3.79 [*P* = .016]), having a total IgE level surpassing the upper limit of the 90th percentile (>798.5 kU/L [OR = 0.37; 95% CI = 0.14-0.98] (*P* = .047)), and having a PLR ≥11.5 (OR = 0.50; 95% CI = 0.24-0.99 [*P* = .050]).

## Discussion

In our retrospective cohort study, we categorized patients with H1AH-refractory CU into 3 clusters based on their treatment responses to omalizumab add-on therapy. Among the 386 patients receiving omalizumab for more 6 months, 40.9% were placed in cluster 1, showing a favorable response marked by a rapid decrease in medication scores and consistent reduction in H1AH doses over 2 years.

Not all patients with CU derive equal benefits from omalizumab therapy. Chuang et al estimated that 61.6% of complete responders, 27.2% of partial responders, and 11.2% of nonresponders among the total of 866 patients with CSU participated in 10 interventional studies of omalizumab.^19^ However, these studies often focused on shorter 12-week or 24-week periods of omalizumab treatment. In contrast, two-thirds of all subjects in our study were treated with omalizumab for at least 12 months. We determined that 37.1% of patients displayed a fluctuating intermediate response, whereas 22.0% consistently had a poor response throughout the add-on treatment. In practice, physicians often adjust omalizumab and H1AH treatments, sometimes increasing H1AH doses or using an OCS during medication reduction periods. This resulted in a higher proportion of patients in the intermediate and nonresponse clusters in our cohort.

Our cohort exhibited trends similar to those reported in clinical trials. The cumulative proportion of patients with H1AH-refractory CU achieving a complete response to omalizumab increased from 55.2% at 3 months to 71.0% at 6 months and 79.8% at 9 months. Our definition of treatment response, although different from clinical trial measures including urticaria activity score, urticaria control test score, and quality of life, yielded similar outcomes. In our alluvial plots, most cluster 1 subjects were early responders, achieving a complete response within 3 months. However, around one-third of early responders were in cluster 2, in which responses fluctuated over a longer period. When we divided the clusters on the basis of omalizumab maintenance duration (<1 year vs ≥1 year), the cluster 2 curves gradually increased up to month 24 for those who continued treatment beyond the first year. This suggests that maintaining omalizumab for more than a year can benefit those who initially achieve a complete response within the first year but later exhibit fluctuating responses. On the other hand, all of the nonresponders identified at the 3-month mark consistently had a poor response throughout the 2-year observation period. These findings suggest that a 3-month omalizumab treatment is sufficient to identify nonresponders. However, for patients showing a favorable response, including OCS discontinuation and reduced H1AH doses during the omalizumab add-on therapy, monitoring for more than 12 months is necessary.

Therefore, our focus is on identifying predictors of nonresponders to omalizumab within the first 3 months of add-on treatment. Previous studies have indicated that low total IgE levels are associated with a lack of response to omalizumab and a slower onset of clinical benefit.^20^^,^^21^ Conversely, higher total IgE levels have been linked to a greater likelihood of achieving a complete response to omalizumab treatment.^19^ However, the specific cutoff values for total IgE level that can reliably predict a response to omalizumab treatment remain a subject of ongoing debate. To this end, total IgE levels around 700 or 1000 IU/mL have been suggested for use as treatment for atopic dermatitis.^22^ Additionally, studies have proposed serum levels of IgE ranging from 15 to 100 IU/mL for predicting a clinical response to omalizumab in CSU.^21^^,^^23^ In our study, patients with CU whose total IgE levels were below the 10th percentile (40 kU/L) were 3 times more likely to be nonresponders than were those with IgE levels of 40 kU/L or higher. Furthermore, elevated IgE levels above the 90th percentile (798 kU/L) in patients with CU independently predicted a delayed response to omalizumab. Simultaneously, initiating omalizumab treatment with doses of 300 mg or higher, along with higher peripheral basophil counts and lower PLRs, were identified as significant factors predicting early responders. Meanwhile, more than half of the patients received omalizumab treatment at a monthly dose of 150 mg. Previous studies have not explored the relationship between high total IgE levels and nonresponse or delayed response to omalizumab, as most have focused primarily on the effects of a monthly 300 mg dose.^19^^,^^21^ As observed in pivotal trials of omalizumab in patients with CU, its effectiveness for managing urticaria symptoms is dose dependent.^10^ Specifically, the 300-mg dose resulted in the highest rates of treatment response. Given the mechanism of action of omalizumab in treating CU, which involves sequestering levels of free IgE and downregulating the FcεRI in mast cells and basophils, it appears that excessively high IgE levels in patients with CU may not be managed effectively by omalizumab at doses of 150 or 300 mg.

The following factors have been reported to be linked to a poor or slow response to omalizumab treatment: type IIb autoimmunity, characterized by positive results in autologous serum skin tests; the presence of IgG autoantibodies against IgE, FcεRIα, and thyroid antigens; elevated basophil histamine release; and increased expression of basophil FcεRI.^11^^,^^21^^,^^24^, ^25^, ^26^ However, conducting these tests is not routine in most real-world practice for patients with CU.

The identification of valid and reliable predictors for treatment responses in CU is currently a prominent area of research. In our study, we reaffirmed that low IgE levels (<40 kU/L) can predict nonresponses to omalizumab add-on therapy after accounting for potential confounding variables.^16^^,^^19^ It is vital to recognize that whereas IgE levels are generally elevated in patients with CSU versus in healthy individuals, low IgE levels, along with basopenia and eosinopenia, are associated with autoimmune CSU, limiting the effectiveness of omalizumab treatment. Additionally, inflammatory markers driven by a combination of neutrophils, monocytes, lymphocytes, and platelets, such as SIRI and PLR, are considered valuable indicators of inflammation in CU as well as in various chronic diseases and malignancy.^17^^,^^27^ Mean platelet volume and platelet distribution width, as markers of platelet activation, have been reported to increase in patients with CU in relation to disease severity.^28^ Recent studies have explored the impact of omalizumab on platelet and inflammatory markers in patients with CU, but these have yielded inconsistent and inconclusive results.^17^^,^^29^^,^^30^ In our study, we observed a notable trend of higher PLRs being associated with a higher likelihood of being a late responder to omalizumab treatment. However, PLR was not found to predict nonresponse to omalizumab. This finding differs from the results reported by Ertas et al,^30^ who noted that nonresponders had a lower platelet distribution width than responders did. In a recent study, pretreatment SIRI was found to be an independent predictor of patients with CSU being omalizumab responders at the 3-month follow-up.^17^ However, in our retrospective cohort with at least 6 months of omalizumab treatment, we were unable to identify SIRI as a significant predictor of being a nonresponder or late responder to omalizumab treatment. Blood basopenia is associated with disease activity, the presence of autoantibodies, and a poor response to H1AH and omalizumab.^31^ Interestingly, our study had similar results, showing that lower basophil counts in the peripheral blood were a significant predictor of a delayed response to omalizumab. To gain a more comprehensive understanding of the potential roles of inflammatory markers in patients with CSU, further studies are needed.

This study has several limitations. First, it had a retrospective cohort design. Thus, specific CU-related information that is typically unexamined in routine clinical practice, such as the presence of autoantibodies, urticaria subtypes, and patient-reported outcome measures, could not be included. Second, the participants were exclusively from a single university hospital in Korea, ensuring a consistent operational definition of clinical response to omalizumab. However, despite adherence to treatment guidelines, variation in the prescription of H1AH, cyclosporine, and omalizumab, potentially diverging owing to practices in other institutions and countries, cannot be dismissed. Additionally, national insurance restrictions for omalizumab use in patients with CU meant that fewer than 40% of the subjects initiated omalizumab treatment at a dose of at least 300 mg per month. This limitation underscores the fact that differences in treatment compliance, whether economically or therapeutically motivated, were not factored into this retrospective analysis. Despite these limitations, we successfully identified and validated the varied effect of omalizumab add-on therapy in patients with H1AH-refractory CU in a real-world setting. This yields invaluable insights into the need for tailored treatment strategies for H1AH-refractory CU.

### Conclusion

In this retrospective longitudinal cohort, adding omalizumab for patients with H1AH-refractory CU proved to be effective, with up to 80% of patients achieving a complete response in 9 months. However, treatment outcomes varied, with low total IgE level (<40 kU/L) predicting nonresponders. The early responders were those who received at least 300 mg of omalizumab and had high basophil counts, IgE levels less than 789 kU/L, and a low PLR. These findings support personalized treatment, with the recommendation that higher-dose omalizumab for high disease activity and elevated IgE levels be administered for more than1 year.

## Disclosure statement

Supported by the 10.13039/501100003725National Research Foundation of Korea funded by the Korea government (grants NRF-2018R1A2B6006199 and NRF-2022R1A2C2006607).

Disclosure of potential conflict of interest: The authors declare that they have no relevant conflicts of interest.

## Figures and Tables

**Supplimentary Fig.1:**