# Supplementary material for: Predicting responses to omalizumab in antihistamine-refractory chronic urticaria: A real-world longitudinal study
Source: J Allergy Clin Immunol Glob. 2024 Mar 19;3(2):100245. doi: 10.1016/j.jacig.2024.100245 (PMC10992700; doi:10.1016/j.jacig.2024.100245)
Supplement: Supplemental Figure [file mmc1.docx]

Supplemental Figure Legend

**Supplemental Figure. 1S.** Treatment response trajectories of omalizumab according to maintenance duration: <1 year (A) or ≥1 year (B)
